# An Eye-Tracking and Forecasting Experiment on Consumer Purchasing Decisions Through Product Reviews

**DOI:** 10.3390/jemr19030064

**Published:** 2026-06-06

**Authors:** Seda Busra Sarac, Kazim Baris Atici, Ismail Bezci, Ata Erinc Dansuk, Fatma Semira Yildirim

**Affiliations:** 1 Department of Business Administration, Hacettepe University, 06800 Ankara, Turkey; sedasarac@hacettepe.edu.tr; 2 Department of Interior Architecture and Environmental Design, Faculty of Fine Arts, Hacettepe University, 06800 Ankara, Turkey; ismailbezci@hacettepe.edu.tr; 3 Presify Analytics, METU Informatics Innovation Center, 06510 Ankara, Turkey; atadansuk@hacettepe.edu.tr; 4 Department of Business Administration, Başkent University, 06790 Ankara, Turkey; fsemirayildirim@baskent.edu.tr

**Keywords:** eye-tracking, consumption motives, purchasing decisions, product reviews, forecasting, machine learning

## Abstract

This study aims to provide insight into consumer purchasing decisions by integrating eye-tracking data with forecasting techniques. First, the study investigates how consumption motives (hedonic vs. utilitarian) and purchasing purposes (for oneself vs. for others) influence visual attention and decision-making processes. An experimental design was conducted with 128 participants in a simulated online shopping environment, where eye-tracking data were collected based on fixation counts and durations across defined Areas of Interest (AOIs). Second, a total of 20 input features were collected, comprising fixation counts and fixation durations for 10 review-related Areas of Interest (AOIs), and these features were evaluated across the experimental scenarios, while the binary output variable represented the participant’s purchase decision. These biometric features, together with scenario information, were used to forecast purchasing decisions using six machine-learning methods, including Artificial Neural Networks, Random Forest, Support Vector Machine, K-Nearest Neighbors, Naive Bayes, and Logistic Regression. The results indicate that consumers’ visual attention aligns with their consumption motives and purchasing purposes, revealing distinct gaze patterns across different scenarios. In the forecasting phase, the accuracy of different methods for predicting purchasing decisions using review-related eye-tracking data is evaluated. Support Vector Machines achieved the highest overall accuracy, approximately 59–60% across the evaluated datasets, compared with a validation-specific majority-class baseline of 53.85%. This corresponds to a modest improvement of approximately 5.15–6.15 percentage points over the naive benchmark. Overall, the findings suggest that objectively recorded review-related eye-tracking data can be operationalized as behavioral input features in a machine-learning-based purchase-decision classification framework, highlighting the methodological value of integrating eye-tracking insights with consumer behavior forecasting.

## 1. Introduction

The study of consumer behavior revolves around understanding the “why, how, where, when, and who” of consumption [1]. It is reasonable to suggest that the primary objective of these questions is to ascertain if and under what circumstances the consumption will take place. Therefore, investigating the behavioral patterns and decision-making processes of consumers when spending their resources on consumption items [2] is of great importance in fulfilling this objective. Indeed, a considerable body of the empirical literature exists on the topic of predicting consumer behavior [3,4,5,6,7] and consumer decision-making process, which starts with consumer needs and thereafter motives [8]. From this perspective, it is obvious that the ability to make inferences about the consumer decision-making process will serve as a key determinant to understanding consumption motives.

Motives can be defined as the processes that guide the behavior of humans [9]. Although motives are also regarded as stimuli that trigger behavior [10], they do not emerge spontaneously unless the relevant triggering factors are present in the surroundings [11,12]. They are based on needs to satisfy and drive people to perceive, consider, and behave in a way conducive to fulfilling a need. This may be a utilitarian need, seeking a useful, practical, and functional benefit, or a hedonic need, characterized by pleasure, enjoyment, and appeal to the senses [9,13,14].

Marketers seek to develop products that respond to needs and activate consumer motives to achieve competitive differentiation [9]. It is crucial, however, to comprehend the underlying motivations driving human behavior, to conduct a meticulous analysis of these factors, and to make efforts in the right direction. An understanding of consumer motivation provides the foundation for a sense of consumer behavior, as well as the sequence of steps leading up to the consumption goal [10].

Furthermore, purchase decisions are principally regarded as the most important product of information processing in the field of marketing [15]. When a consumer has a certain purpose for purchasing, first they analyze the scenario, when and what they are purchasing as well as whom they are making the purchase decision for. During those stages they get affected by both marketing activities conducted and environmental stimuli [16], such as their own experiences with the product, what kind of purchase channel is used, or whom the purchasing decision is to be made.

In marketing research, diverse measurement techniques are employed to assess consumer responses to stimuli. Among these, conventional self-report measures, including questionnaires, interviews, and focus group discussions, are the most prevalent [17,18]. While these methods offer insights into consumer preferences, they may fail to capture the full context of consumer interaction with marketing stimuli. Additionally, self-report data can be prone to incompleteness due to memory limitations and biases [18]. At this point, cognitive or psychological data might help the data-gathering process to better grasp the full context of an event [19], which could result in more accurate forecasts.

Integrating cognitive or psychological data into the analysis of consumer behavior practices is currently associated with the field of *neuromarketing*, which refers to the field of research used to refer to the utilization of neuroscientific methods for the analysis and understanding of human behavior within the context of marketing activities [20]. The primary methods employed within this research paradigm are neuroimaging techniques and biometric measures, including eye-tracking (ET). These allow researchers to overcome the limitations of conventional techniques by capturing consumers’ unexpressed cognitive and psychological (emotional) states in reacting to marketing stimuli [17,20,21]. For example, while traditional methods such as manual observation or headcams may be sufficient to identify the customer’s stops, paths, head movements, or gaze direction, they lack the ability to measure visual attention, which is considered a significant prerequisite for most shopping decisions. The ability to ascertain customer attention with such precision is made possible only by eye-tracking technology. It is capable of identifying the almost exact location of customers’ gaze, discerning where they are directing their attention, and furnishing detailed insights through scan paths, or metrics on fixations, and saccades [22]. This enhanced understanding can lead to more effective marketing strategies and informed business decisions like designing websites and package elements or search optimization [23,24,25]. Recent neuromarketing research has similarly used eye-tracking to evaluate how visual components of digital marketing materials influence purchasing-related responses [26]. To move beyond understanding, forecasting methods can be beneficial to facilitate forward planning and the making of more rational business decisions [27].

In this research, we aim to utilize an experimental design to contribute to eye-tracking research in marketing. The primary objective is to provide an understanding of consumer purchasing decisions by examining gaze patterns (fixation duration and count) on product reviews during the purchasing process and to forecast consumer purchasing decisions through eye-tracking data. Specifically, research objectives are threefold and interrelated:

- ***Enhancing our understanding of the cognitive processes underlying consumer decisions based on product reviews.*** We seek answers to the question of how consumption motives (hedonic and utilitarian) and/or purchase purposes (purchasing for oneself and others) influence the direction of attention during the decision-making process.
- ***Forecasting purchasing decisions.*** We aim to offer an approach for forecasting purchasing decisions by integrating eye-tracking metrics.
- ***Experimenting with different forecast methodologies.*** We examine the relative performance of six machine-learning methods in forecasting purchase decisions: Artificial Neural Networks, Random Forest, Support Vector Machine, K-Nearest Neighbors, Naive Bayes, and Logistic Regression.

For this main objective, we present an experimental design with *varying motives (hedonic and utilitarian)*, *varying purchasing purposes (purchasing for oneself and others*), and *varying forecasting methods*. To the best of our knowledge, there is no study that utilizes eye-tracking data to make forecasts on consumer purchasing decisions based on their consumption motives and purchasing purposes. This study presents a novel approach to gain insights into consumer behavior in the context of diverse consumption motives and purchase purposes through the utilization of eye-tracking data and forecasting techniques.

The paper is organized as follows. Section 2 reviews the general tendencies in the research streams that have close ties to the current research design: consumption motives, purchase purposes, online shopping behaviors, and eye-tracking research. Section 3 presents the methodology and explains the details of the eye-tracking experiment along with the forecasting methods utilized. In Section 4, the findings and insights derived from both eye-tracking and forecasting analysis are presented. Finally, Section 6 concludes the paper.

## 2. Theoretical Background

The theoretical background of the current research can be framed around several interrelated topics such as *hedonic and utilitarian consumption motives*, *purchase purpose*, *basics of eye-tracking applications in marketing*, *online shopping and the utilization of product reviews* and *forecasting through eye-tracking research*, respectively. In this section, we briefly present the fundamentals of the main concepts leading to the current research as well as the reflection of these concepts in eye-tracking research.

### 2.1. Consumption Motives: Hedonic and Utilitarian

Throughout history, motives have been well-researched by behavioral scientists in different forms such as *Maslow’s Theory of Need Hierarchy*, *Alderfer’s Model*, *Herzberg’s Two Factor Theory*, *Process Theories*, and *Equity Theory*. In marketing research, motives are regarded as a pertinent concern and are often investigated, much like in other fields (such as psychology, and management) where they are a pertinent issue. The rationale is that a fundamental component of marketing strategies lies in understanding motives and implementing this understanding into effect [28,29]. Indeed, the marketing process begins with understanding marketplace and consumer needs and wants [30].

Consumption motives can be described as the rationale for a purchase action that is based on the type of satisfaction that the consumption will provide [31]. Therefore, they directly influence how consumption behavior is shaped. The motives underlying consumption are diverse and multifaceted in nature, with influences that include utilitarian, hedonic, social, and psychological factors. The literature on consumption motives has shifted towards a binary categorization system, which evaluates the degree of the sensory attribute associated with decision-making behavior [32]. Hedonic and utilitarian motives are two of the primary consumption motives. Hedonic motives are associated with sensory attributes such as pleasure, enjoyment, and satisfaction. Utilitarian motives, on the other hand, are linked to non-sensory attributes such as usefulness, practicality, and efficiency. The motive behind the purchasing of a product to be classified as either hedonic or utilitarian is a reflection of its relative attributes within the context of consumer experience [33]. This categorization is not always mutually exclusive. Many products possess both hedonic and utilitarian attributes, leading to varied consumer interpretations. For instance, a chocolate bar can be perceived as a hedonic product when consumed in a pleasure-oriented setting, or as a utilitarian product when consumed in a more goal-oriented setting such as to satisfy a nutritional need or complete a task [34].

### 2.2. Purchase Purpose: Purchasing for Oneself or Someone Else

The idea of compartmentalizing the decision-making process for purchasing is suggested by Kotler in 2001 as the five stages of the purchasing decision process, which are: *problem recognition*, *information research*, *evaluation of alternatives*, *purchase decision*, and *post-purchase behavior*. Kotler’s [16] framework is typically used in the literature to discuss purchasing decisions. The viewpoint of the person for whom the purchase is intended is very important because steps in the decision-making process can move more slowly when there is a greater degree of risk involved, which is likely to happen when the purchase is for someone other than yourself [35]. The purchase decisions made for someone else can be interpreted as high-involvement purchase decisions due to the higher risk [36,37]. Choi et al. [38] revealed in eye-tracking research that when the level of involvement is higher for the consumer, i.e., when the involved risk is higher, the required time and the number of factors considered for purchasing decisions is higher compared to the low-involvement counterpart.

### 2.3. Online Shopping and Utilizing Product Reviews

Online shopping has been an important subject of discussion in marketing studies for many years starting from the internet’s rise to popularity. In line with that, analyzing the online shopping behavior of consumers is of great interest in the marketing literature. A recent review of 197 research papers in the area by Singh and Basu [39] reveals that 47% of the online shopping behavior research papers focus on *user behavior.*

While online shopping makes it possible to justify a purchase by offering helpful information and serving as a handy channel for consumption, it can serve as an important source of data for marketing research. One of the beneficial sources to understand the motives behind purchase decisions are the product reviews on online shopping sites or applications. According to Maslowska et al. [40], product reviews are one of the most focused elements in an online shopping channel.

Since product reviews possess a strong potential to disclose consumer motives, they unavoidably turned into an important subject of research. This is reflected in eye-tracking research attempts. It is possible to find research on product reviews that try to attach reviews to hedonic and utilitarian motives. For instance, Chen et al. [41] categorize online shopping product reviews into attribute-based reviews (ABRs), which have utilitarian properties, or experience-based reviews (EBRs), which have hedonic properties. They reveal ABRs and EBRs contribute to the perceived helpfulness more when the corresponding consumption goal (utilitarian or hedonic) given to the consumers are compatible. In this context, the Review Helpfulness Theory offers a valuable framework to understand why such consistency is important. This theory states that consumers do not evaluate reviews solely based on content quality but also on the relevance of the review to their current consumption motives [42]. Further exploring the product reviews, in eye-tracking research, Luan et al. [43] state review type, in the context of which consumption goal it is associated with, is not very significant when consumers are shopping for utilitarian products and supported this finding by having similar outcomes with several different products. When the product is clothing, which is classified as an experience-based product, they observed that consumers gazed the ABRs even more. Whereas for hedonic products, it was observed that attention tends to be on EBRs as expected. Furthermore, Guan and Lam [44] utilize product ratings as important factors in understanding the consumer purchasing decisions and emphasize the investigation of motives when reading the product reviews as one of the promising further research avenues. In a further study by Jin et al. [45], an eye-tracking analysis of online reviews was conducted in the context of the framing effect, leading to the conclusion that the summary descriptions of online reviews for utilitarian products garnered longer fixation durations than those for hedonic products. These fixations predominantly occurred on the functional attributes of the product in question.

### 2.4. Basics of Eye-Tracking Applications in Marketing

One of the main interests of *neuromarketing* is to integrate cognitive or psychological data into the analysis of consumer behavior. It is closely associated with *neuroscience*, which is a form of understanding human behavior using the nervous system of people as a biological factor. Shiv et al. [46] point out that neuroscience sheds light in the context of decision-making by providing much-needed evidence for proposed theories to understand the underlying processes of decision-making, as well as refining or giving new alternatives to existing proposals. Closely related to this mission of neuroscience, neuromarketing tools have the potential to be effective in understanding the decisions made by consumers through cognitive data. One of the most common methods of neuromarketing is the use of eye-tracking [47].

Eye-tracking research typically involves presenting stimuli, such as videos or photographs, to test subjects while simultaneously recording their pupil and corneal movements. This method offers several advantages over conventional methods like questionnaires or self-reports, particularly in behavioral studies. One key benefit is the reduction of errors that can occur when subjects struggle to recall the details of the experiment accurately [48]. Additionally, eye-tracking allows researchers to understand how visual stimuli impact consumer attention. Accordingly, eye-tracking has the potential to infer their preferences without the necessity for explicit responses. Furthermore, eye movements can reveal unconscious preferences that participants may prefer not to disclose in self-reports [49]. For instance, it is proven that when a consumer has a higher visual focus on an advertisement, the likelihood of a positive effect occurring from that advertisement increases [50].

More specifically, eye-tracking metrics such as fixation duration—the total time spent on a given Areas of Interest—and fixation count—the number of distinct gaze fixations within that area—serve as critical indicators of cognitive processing and interest [51]. Recent research has shown that longer fixation durations are associated with increased cognitive effort and deeper processing of visual information, indicating higher levels of consumer engagement [52]. Similarly, higher fixation counts are positively correlated with stimulus relevance and decision-making intensity, particularly in contexts involving product reviews and advertising exposure [53]. In the context of consumer behavior, longer fixation durations and higher fixation counts on particular products or visual elements suggest higher levels of attention and deeper information processing. These are often associated with the formation of preference and purchase intention. Glaholt et al. [49] suggest that eye movement data, especially fixation metrics, can effectively predict preferences in decision-making tasks. These metrics enable researchers to move beyond merely identifying which stimuli attract attention to understanding how such attention translates into decision-making [54]. Consequently, fixation-based metrics are essential for making reliable inferences about consumer preferences in visual marketing contexts.

### 2.5. Forecasting with Eye-Tracking Data

For many years, forecasting *the act of predicting and estimating future events and trends based on the information at hand* [55] has been a subject of interest for scholars from a wide range of disciplines. In the business discipline, forecasting attempts constitute a great body of research especially in production, finance, and marketing. While forecasting approaches are common in predicting market movements in finance, within marketing research, there exists a wide body of research focusing on forecasting demand [56,57,58,59].

Forecasting researchers frequently employ historical data related to the feature(s) they are attempting to predict as well as previous values of those feature(s). For instance, in the context of stock price prediction, researchers may utilize historical data such as stock market index values, currency exchange rates, and interest rates [60]. Similarly, those working on electricity price forecasting often incorporate demand data and historical price information [61]. In the field of virtual reality, researchers studying gaze-contingent rendering use eye-tracking data to inform their models [62]. Furthermore, there are unconventional forecasting methods such as predicting stock market movements using social media posts [63] or using eye-tracking data to predict the effects of socializing on decision-making [64].

Eye-tracking can provide insight into a person’s internal thought process [64] which can help to predict the patterns between eyes and the choices that are being made. Utilizing eye-tracking data to predict consumer behavior and preferences during shopping is an interesting research area in the marketing literature. For instance, Glaholt et al. [49] used eye-tracking data to predict preferences the participants had about logos and the faces they were being shown. Imai et al. [65] use a combination of eye-tracking and mouse movements to determine how much the participants overstate their desire to purchase a product. Mikalef et al. [66] make use of eye-tracking data of the participants to predict which product they will choose from and obtain clues about which parts of information the participants gave more importance during their decision-making. Recent studies have also explored AI-powered eye-tracking approaches in online review contexts, further indicating the relevance of visual attention metrics for understanding consumer perceptions and purchase-related behavior in digital environments [67].

Relying on our review above, in the current research, we bring *consumer motives* and *purchasing purposes* together in an *online shopping* experience that incorporates *product reviews*. *An eye-tracking* experiment is designed to collect biometric data of participants, and the data is utilized to *forecast* purchasing decisions. The following section provides the details of our data and methodology.

## 3. Materials and Methods

The current research aims to investigate the potential of eye-tracking data for forecasting purchasing decisions. Accordingly, the methodology of the research is composed of two phases. The first phase is an eye-tracking experiment to collect biometric data of participants during an online shopping experiment. In the second phase, the data collected in the experiment stage were used to train and test different algorithms of machine learning to forecast purchasing decisions.

### 3.1. Eye-Tracking Experimental Design and Procedure

The eye-tracking experiment is based on an online shopping experience for purchasing a chocolate bar. To eliminate the effects of participants’ previous experiences, a non-existent chocolate packaging and branding (*ChocoPist*) with an online shopping site interface including product reviews were designed for the experiment. The participants were not provided with any description of the experimental content either in visual or written format before the experiment. Before data collection, approval was taken from the University Ethics Commission (Approval Number: E-66777842-300-00003489417).

**Study 1. Scenarios and Manipulation Check**

***Scenarios.*** We have two different motives for the products: utilitarian and hedonic. Also, the purchasing purpose is differentiated as purchasing for oneself and purchasing for others. The combinations of these (two different consumption motives and two different purchase purposes) constitute four different scenarios of purchasing as given below:

**Scenario 1 (Utilitarian and for oneself).** *You are on the school volleyball team. Before training, you will buy a chocolate bar to provide you with the energy you will need during physical activities, to increase your energy level and performance.*

**Scenario 2 (Utilitarian and for someone else).** *You will buy a chocolate bar to provide the students in the school’s volleyball team with the energy they will need before training sessions, and during their physical activity, to increase their energy levels and performance.*

**Scenario 3 (Hedonic and for oneself).** *You are going to the match to support your friends on the school volleyball team. You will buy yourself a chocolate bar to snack on while watching the match.*

**Scenario 4 (Hedonic and for someone else).** *Students who want to support their friends on the school volleyball team are going to the match. You will buy a chocolate bar for the students to snack on while watching the match.*

***Validation*.** Before moving to the eye-tracking experiment, to validate the scenarios for consumption motives (hedonic and utilitarian) an online survey that consists of six items borrowed from Choi et al. [13] was conducted after the necessary permissions were obtained from the corresponding author. Each scenario was evaluated by randomly assigned 50 participants (200 participants in total). For every scenario, the participants evaluate 6 items representing utilitarian (useful, practical, and functional) and hedonic (pleasant, fun, and enjoyable) points of view with a five-grade bipolar scale (e.g., 1: not useful, 5: useful). Note that the participants of this preliminary test were different from those who will be participating in the eye-tracking experiment. *T-tests* are applied within scenarios and the results reveal that there is a significant difference between scenarios. Relevant *p*-values are provided in Table A1 of the Appendix A.1.

**Study 2. Eye-Tracking**

***Apparatus.*** The eye-tracking experiment was conducted in a *Business Analytics & Decision Sciences* Lab of a public university in Turkey. The lab meets the requisite conditions for accuracy (light level, sound insulation, etc.). The *Tobii Pro Fusion* (produced by Tobii AB, Sweden) eye-tracking device featuring a dual-camera eye tracker operating at a 60 Hz sampling rate is utilized. The eye-tracking data were recorded using a 27-inch monitor (3840 × 2160 pixels) to display the stimuli. In the pre-experimental phase, participants were positioned at a distance of approximately 60 cm from the monitor. Before the commencement of the experiment, a standard in-built 5-point calibration and 4-point validation procedure was implemented to calibrate the participants’ eyes. This procedure was considered successful when the eyes were calibrated to an accuracy within 1° of the visual angle.

***Participants.*** The eye-tracking experiment sample consists of undergraduate students, graduate students, and academic staff. The participants were invited to the experiment directly by the researchers, both via invitation letters published on social media platforms, face-to-face conversations, and through conversational interactions conducted in person. In response to the invitation, 161 potential participants expressed interest. Among them, 152 individuals (87 females and 65 males) enrolled voluntarily in the experiment. 15 participants were excluded due to unsuitable calibration results. Additionally, 9 participants (five female and four male) who completed the experiment with gaze sample acquisition rates below the 80% threshold were also excluded from the data. Consequently, the final sample consisted of 128 participants with normal or corrected-to-normal vision and full-color vision, all of whom provided valid eye-tracking data. The participants were aged 18–42 years, with a mean age of 22.6 years (SD = 4.61). Since participants were recruited from undergraduate students, graduate students, and academic staff through invitation-based participation, the resulting sample can be characterized as a convenience sample. To ensure the consistency and comparability of the experimental results, participants were randomly assigned to one of four scenarios, with equal distribution across all. The details of the final sample profile are given in Table 1.

***Assignment.*** Each participant was randomly assigned to one of these four scenarios. Overall, the participants were evenly distributed between scenarios. Each participant saw the same screen consisting of a chocolate bar and product reviews about it. The reviews were prepared specifically for this research. Each review reflects hedonic and utilitarian consumption motives and is produced by consulting with a marketing expert. The word count was kept almost constant (25 ± 1) throughout the reviews in the original version. This is due to maintaining a consistent and moderate word count which has been demonstrated to enhance processing fluency and increase the perceived informativeness of a review, thus improving its overall helpfulness [41]. English translations of all reviews are provided in Table A2 of the Appendix A.2. The examples that reflect utilitarian and hedonic motives’ product reviews are given below:

*Utilitarian—Thanks to the 8.1 g of protein it contains, one pack assuages your hunger. It’s a bit high in fats but it contains no trans-fat.*

*Hedonic—With its unique style which makes one think of handmade chocolates, ChocoPist is indispensable to those who want to make themselves and (lucky) others special!*

***Stages of the experiment.*** Before initiating the experiment, a preliminary information video was shown to the participants outlining the details of the experimental process and explaining the steps to be followed during the experiment. Then, the participants were taken to the experiment in the laboratory (in an isolated area providing the necessary physical conditions for the experiment). Furthermore, brief information was given, and written informed consent was requested.

**Figure 1:**
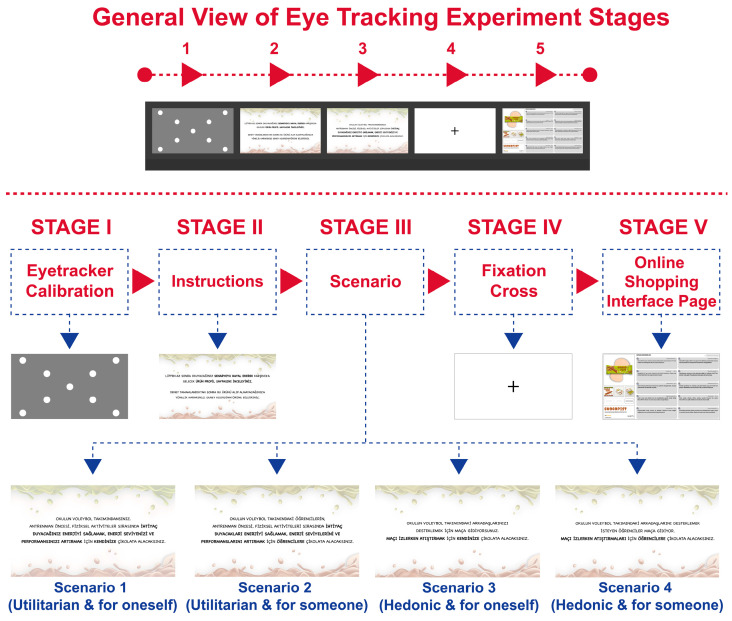
Eye-tracking experiment design. The figure displays the original text that the participants are exposed.

The experiment is conducted in five stages explained below. The experimental design is also illustrated in Figure 1.

**Stage 1.** The experiment starts with eye-tracker calibration. (Note that the participants whose calibration results were not appropriate were not included in the experiment).

**Stage 2.** Following calibration, an information text describing the experimental process and instructions are shown. The instructions, which had been previously outlined verbally to the participants, were then presented in written form for a second time. These instructions are to examine the online shopping interface page in line with the scenario to be given to them and to report their decision about whether or not to purchase the product at the end of the experiment.

**Stage 3.** Following instructions, the participant sees one of the four scenarios given above in a text form.

**Stage 4.** Before the stimulus (online shopping interface), a blank page with a plus sign in the middle of it (fixation cross) was shown to the participants to eliminate the differences that could occur from the starting coordinates of the gaze samples. After fixation cross, the online shopping interface with the product image and reviews that can reflect utilitarian and hedonic motives (5 each) were shown to the participants. The stimulus was shown to each participant for 50 s, which was determined after several pilot experiments which were not included in the final sample.

**Stage 5.** Following the completion of the eye-tracking task, the participants were invited to indicate whether they would be purchasing the product in adherence to the scenario that had been provided to them. Their decision was recorded. Upon completion of the experimental process, participants were given a debriefing session, during which the purpose of the experiment was clarified and they were encouraged to ask any questions they might have.

**Figure 2:**
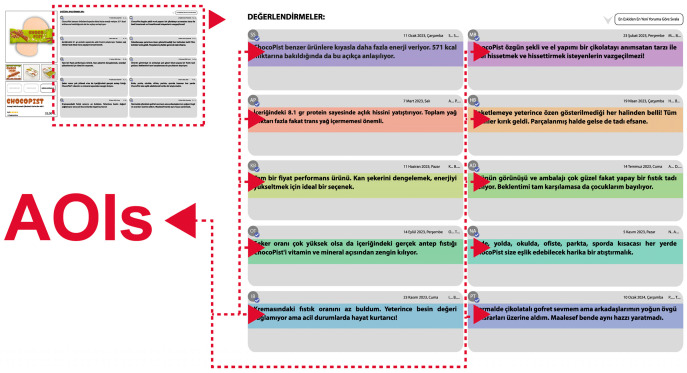
Areas of Interest. The figure displays the original text that the participants are exposed.

***Areas of Interest (AOIs).*** In eye-tracking experiments, AOIs refer to parts in a stimulus that were selected according to the purpose of the study for ease of data analysis. Usually, the main stimuli for the experiment are later segmented into Areas of Interest (AOIs) in order to exclude unrelated parts of the stimuli (e.g., blank spaces). In our experiment, the product reviews on the online shopping page were prepared with mock dates and fake personal information for each review to match the appearance on real websites. It was observed that these details also attracted some attention from the participants. For our analysis, since the main focus is on product reviews, the parts of the online shopping screen that include review text are selected as our AOIs (see Figure 2) and, therefore, person and date details for review texts are excluded so that the forecasting phase solely focuses on reviews. Furthermore, product images, packaging, price, and other non-review webpage elements were excluded as AOIs in the forecasting dataset. This choice is indicative of the review-centered scope of the forecasting analysis, rather than an assumption that such visual elements are irrelevant to consumer attention or purchase decisions.

Ten review-related AOIs were identified. Fixation count and fixation duration metrics were extracted for each selected AOI from *Tobii Pro Lab* (v.1.241.54542) biometric research software. Fixations refer to *the pauses we make while gazing at a stimulus that enables us to process the information* [62]. For the forecasting dataset, fixation data were aggregated at the participant-AOI level by calculating total fixation count and total fixation duration separately for each participant and for each of the 10 review-related AOIs.

### 3.2. Forecasting Methods

The forecasting phase was intentionally designed as a review-based purchase-decision forecasting task. The objective of the study was not to model purchase decisions using all visual elements of the online shopping interface, but rather to examine whether visual attention allocated specifically to user-generated product reviews provides predictive information about binary purchase decisions. Therefore, the forecasting dataset was restricted to AOIs related to reviews. At the end of the eye-tracking experiment, we ended up with a dataset of 128 participants’ fixation counts, fixation durations, and purchasing decisions, as well as categorical features of the *scenario assigned (1 to 4)*, which intrinsically contain *consumption motives (hedonic* vs. *utilitarian)*, *purchasing purpose (purchase for oneself and purchase for someone else)*. During the experiments, the participants were offered two options to indicate their purchasing decision: to purchase the product or not. Therefore, the target variable for our forecasts is a binary (purchase: 1, do not purchase: 0).

Machine-learning models offer various advantages depending on the context. Each forecasting method serves different purposes and performs differently based on the data and expectations. While no single method can be deemed universally superior, the existing literature provides insights into preferred approaches, especially in demand forecasting. According to a review by Aamer et al. [68], Neural Networks (49%) and Support Vector Machine/Regression Models (27%) are the most commonly used across several industries. Random Forest, K-Nearest Neighbors, and deep learning methods are also notable. Similarly, Anitha and Neelakandan [69] highlight the effectiveness of clustering, classification, regression, and deep learning models in demand forecasting. Note that our input data consists exclusively of quantitative biometric metrics (fixation counts and durations) rather than textual data. Given the structured nature of the eye-tracking dataset and the sample size, traditional machine-learning algorithms (such as SVM, RF, and ANN) were selected as the most appropriate and robust baselines, consistent with established practices in demand forecasting and the eye-tracking literature. Based on this literature and practical considerations, the forecasting phase of this study employed the following machine-learning-based techniques:

***Artificial Neural Networks (ANNs).*** ANNs are machine-learning models that take inspiration from the structure of the human brain [70]. The human’s ability to succeed at cognitive–perceptual tasks shows that large neural networks are capable of many computationally demanding tasks and actions [71]. The processing elements of ANNs are called nodes and these nodes are interconnected. The connection weight between the nodes corresponds to the synapse, the link that connects neurons in the human brain. The transfer function in the nodes allows an ANN to be able to have nonlinearity in its computations and changing the connection weights between the nodes gives ANNs the capability to learn. Their aptness to work with incomplete or noisy data and their ability to learn from mistakes and recognize patterns make ANNs popular in many fields and these qualities also gather the attention of researchers who are interested in forecasting.

***Random Forest.*** It is a machine-learning method that can be applied to regression and classification problems. Random Forest consists of multiple decision trees and can be applied to both regression and classification problems. The result is the option selected by the most decision trees [72]. The Random Forest algorithm can help mitigate overfitting problems [73], and using multiple decision trees instead of a single decision tree reduces the issue of poor generalization [74].

***Support Vector Machine (SVM):*** It is a supervised learning method in machine learning that can be used for classification and regression tasks. SVMs use linear hyperplanes to separate the space the data points are in and choose the hyperplane that provides the highest margin to obtain the best decision boundary that separates two classes. Although the hyperplane used by SVM is linear, it can be used to solve nonlinear classification tasks with the kernel method where data is transformed into multidimensional feature vectors and it becomes possible to linearly separate them [75].

***K-Nearest Neighbors (KNN):*** It is a non-parametric classifier [76]. To classify a data point its k nearest neighbor data points are looked at and according to the majority vote in that data neighborhood, the data point is appointed a class label. Since the k value, which determines how many data points will be checked during the classification, affects the performance of the classifier, it is important to determine an appropriate K value [77].

***Naive Bayes:*** It is a type of classifier that works with the principles of probability after making a few important assumptions. It assumes that the features are conditionally independent and it also assumes all features contribute equally to the classification [78]. While these assumptions are often not correct in real life, Naïve Bayes classifiers perform well in many real-life problems [79].

***Logistic Regression:*** It is one of the probabilistic classifiers used in supervised learning. To calculate the probability of a data point belonging to a class, a weight is given to the features of the data which determines how important each feature is for the classification task. A bias term is added to the weighted features and the resulting number is put through a sigmoid function, which maps the numbers between 0 and 1. To use Logistic Regression as a binary classifier, a cut-off point such as 0.5 can be selected on this function to create a decision boundary that determines whether a data point belongs to a given class [80].

## 4. Results

The findings of the current research can be undertaken in two parts. The first set of findings consists of the observations from the eye-tracking experiment. In the subsequent part, we present the findings of forecasting models utilized with the data extracted from the eye-tracking experiment.

### 4.1. Observations in Eye-Tracking Experiment

**Figure 3:**
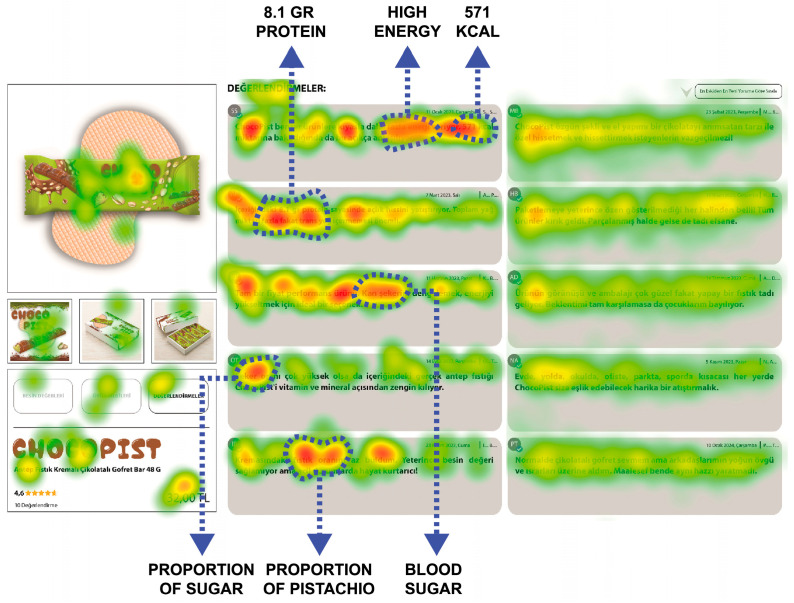
Heat map for participants in Scenario 1 (utilitarian and purchasing for oneself). The figure displays the original text that the participants are exposed.

**Figure 4:**
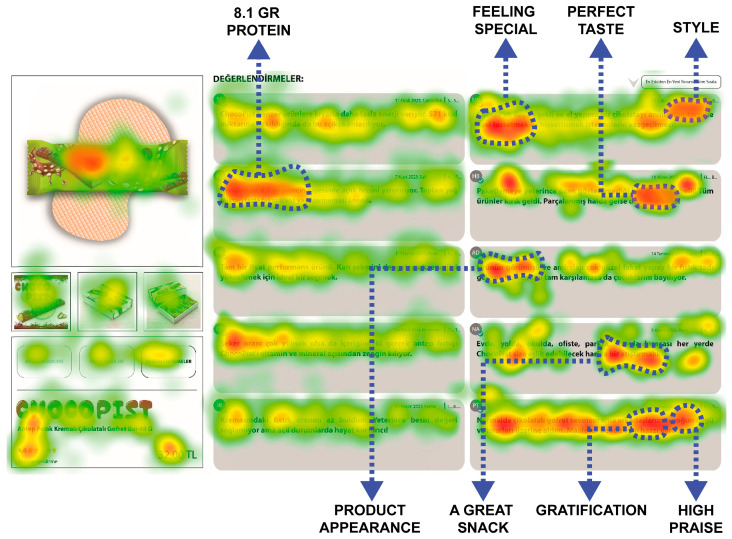
Heat map for participants in Scenario 3 (hedonic and purchasing for oneself).

Before extracting the data out of the eye-tracking for forecasting, the recordings were visually examined in the *Tobii Pro Lab* biometric research software to identify any significant findings or patterns. The below observations are extracted from eye-tracking recordings:

*Observation 1.* In the scenario provided, the participants demonstrate a greater tendency to attend to product reviews that align with their consumption motives, exhibiting longer fixations compared to other reviews. For instance, those who examine a utilitarian scenario tend to direct their attention more toward attribute-based parts of the product reviews about the nutritional value of the product. On the other hand, those who examine a hedonic scenario tend to direct their attention more toward experience-based parts *(such as feeling special, a great snack, perfect taste,* etc.*)* of the reviews. Examples can be observed in the heat maps given in Figure 3 and Figure 4 below.

**Figure 5:**
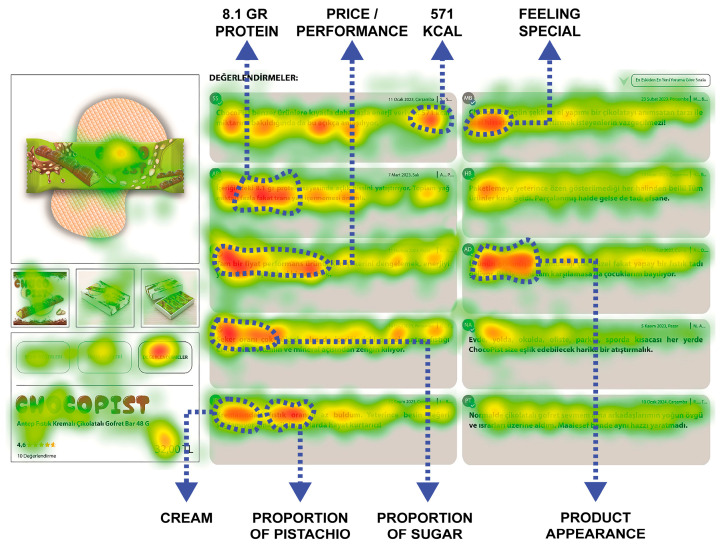
Heat map for participants attending Scenario 2 (utilitarian and for someone else).

**Figure 6:**
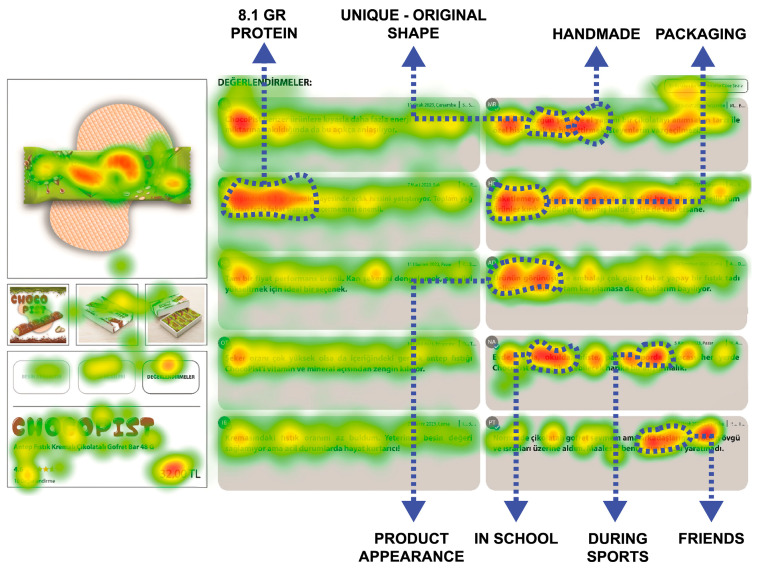
Heat map for participants attending Scenario 4 (hedonic and for someone else).

*Observation 2.* Regardless of whether the participants make the decision with hedonic or utilitarian motives when they are purchasing for someone else (Scenario 2: utilitarian and for someone else; Scenario 4: hedonic and for someone else), in addition to the nutritional values, specific attention has been observed to keywords that could be attributed to hedonic motives such as *feeling special, handmade chocolate*, *unique-original shape*, *in school*, *during sports*, *friends as well as the appearance of the product*. These can be observed in the heat maps presented in Figure 5 and Figure 6.

**Figure 7:**
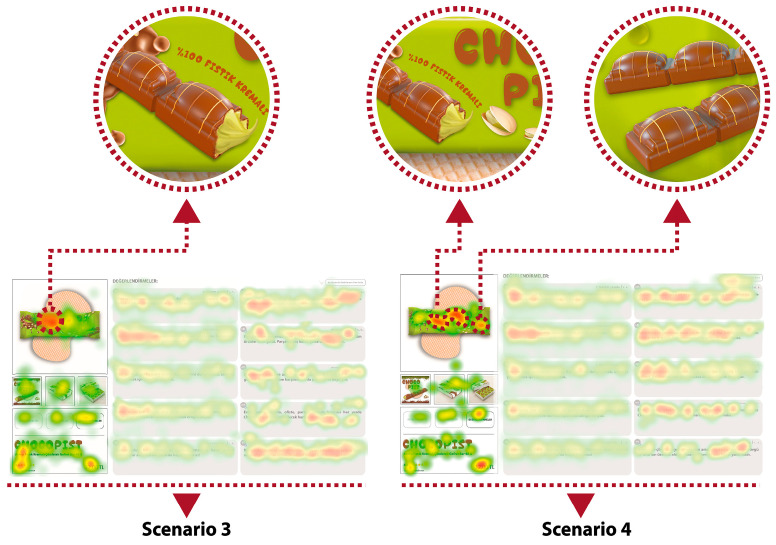
Heat map for participants attending Scenario 3 and Scenario 4 (hedonic Scenarios).

*Observation 3.* The protein content of the product was a notable keyword for almost all participants, regardless of the scenario they were given (see Figure 3, Figure 4, Figure 5 and Figure 6 above). On the other hand, the heat map-based visual analysis suggests that participants assigned to hedonic scenarios (Scenario 3 and Scenario 4) also tended to allocate visual attention to the appearance of the product on the packaging (see Figure 7 for the heat map).

**Figure 8:**
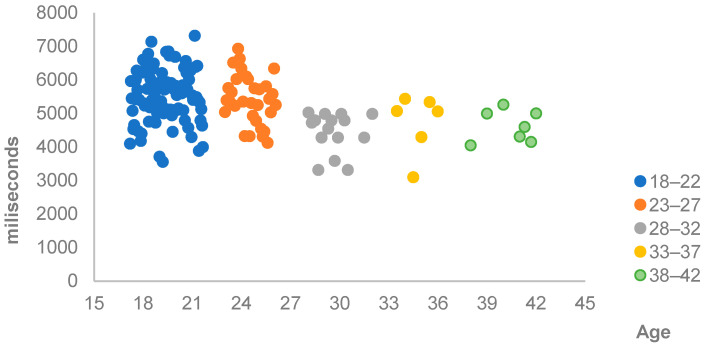
Total durations of fixations of all participants.

*Observation 4.* A holistic examination of the gaze data from all participants, across all scenarios and purchase purposes, revealed that participants in the 18–22 and 23–27 age groups exhibited longer total fixation durations compared to those in other age groups. A graph of the total durations of fixations for each participant is presented in Figure 8 below.

To sum up the above observations, it is evident that participants, generally, examine product reviews in line with their relevant consumption motives and allocate their attention in line with their purchase purposes. Additionally, consumers making a purchase for someone else tend to put additional focus more on specific keywords in product reviews that align with hedonic motives. Moreover, observations derived from heat maps suggest that participants assigned to hedonic scenarios may also attend to product packaging. It should be noted that these packaging observations are derived from a qualitative heat map inspection and are distinct from the subsequent forecasting analysis, which focuses specifically on review-related AOIs. Finally, younger participants tend to have longer fixation durations.

### 4.2. Forecasting Phase

The forecasting analysis was conducted in Python (version 3.12.8). Following data exportation from the Tobii Pro Lab biometric research software, the data were imported from Excel files and processed in Python. The scripts employed in the analyses have been made publicly available on GitHub (v.3.5.12): https://github.com/badslab-collab/consumption-motives (accessed on 30 May 2026).

The full dataset consists of 21 input variables and 1 output variable associated with 128 participants: *fixation counts of 10 review-related Areas of Interest (AOIs), fixation durations of 10 review-related Areas of Interest (AOIs)*, and the *assigned scenario (1 to 4)* as inputs and *purchasing decisions (0–1)* as the output. As the forecasting phase focuses on review-based visual attention, product image and packaging-related gaze metrics were not included as predictive features in this dataset. We utilized three datasets with different features and six machine-learning methods—*Artificial Neural Networks (ANN), Random Forest, Support Vector Machine (SVM), K-Nearest Neighbors (KNN), Naïve Bayes and Logistic Regression*—for each dataset to obtain purchasing decision forecasts. The target variable for all models is the purchasing decision and it is a binary variable (purchase: 1, do not purchase: 0). Before constructing the forecasting dataset, the eye-tracking data were screened based on predefined data-quality criteria. Participants with gaze sample acquisition rates below 80% and those whose calibration/validation accuracy error exceeded 0.9° were excluded from the dataset. No additional outlier removal or normalization procedure was applied after this eye-tracking data-quality screening.

The dataset, derived from our several-minute-long, in-person eye-tracking experiment involving 128 participants, represents a robust sample size within the context of conventional eye-tracking research [81]. Nonetheless, in the domain of machine learning, substantially larger datasets are generally required to mitigate the risk of overfitting. To address this issue, studies using eye-tracking data, such as ours, typically employ validation methods, including k-fold cross-validation and Monte Carlo validation. K-fold cross-validation refers to partitioning a dataset into k disjoint subsets and iteratively using each subset for validation [82]. Monte Carlo validation, on the other hand, involves splitting the dataset into two subsets at a predetermined ratio and repeatedly evaluating the model across a predefined number of iterations [83]. Previous studies have shown that these approaches are effective in reducing overfitting when applying machine-learning techniques to small-scale eye-tracking datasets. For example, Wang et al. [84] applied machine learning to a relatively small eye-tracking dataset derived from a controlled laboratory experiment by using a k-fold cross-validation procedure that protected their model against overfitting and ensured stable and reliable performance estimates for predictive models. To conduct a robust and comprehensive validation of our models, we applied both 10-fold cross-validation, which is deemed sufficient in the literature [83], and Monte Carlo validation with 200 repetitions for all models except the ANN. In these models, the two procedures were integrated by performing a full 10-fold cross-validation within each Monte Carlo sampling iteration, thereby providing a multilayered assessment of model generalizability. However, we adopted a different validation strategy for the ANN model due to its statistical properties. Artificial neural networks are highly parameterized models and are sensitive to the size of the validation set. When k-fold cross-validation is applied to small datasets, increasing the number of folds can reduce the size of the validation sample, leading to unstable performance estimates. In contrast, Monte Carlo cross-validation does not suffer from this limitation, since it evaluates model performance through repeated random sampling while keeping the validation set size fixed, thereby providing more stable error estimates for artificial neural network models [85]. Therefore, 10-fold cross-validation was not implemented for the ANN; instead, the model was evaluated exclusively through Monte Carlo validation with 200 repetitions, using repeated random sampling as the validation strategy.

The same train–test partitions were used across all machine-learning algorithms to ensure fair comparison, reproducibility, and controlled model evaluation. This design choice prevents performance differences from being driven by different random data splits and allows the observed differences to be attributed more directly to the algorithms themselves. Accordingly, the validation-specific majority-class baseline was identical across models within each dataset. In this study, this baseline was calculated as 53.85% and used as the naive benchmark for interpreting model accuracy.

Regarding model specification, hyperparameter optimization was not performed; instead, the models were implemented using standard or near-default parameter settings. This choice was made to avoid adding further model-selection complexity to a relatively limited experimental dataset, as hyperparameter tuning on small samples may increase the risk of overfitting during model selection [86]. Therefore, standard parameter settings were preferred to support model generalizability.

In addition to hyperparameter settings, class distribution was also considered before model estimation. Class imbalance handling methods were not applied. The dataset included 78 positive and 50 negative purchase-decision observations, and this imbalance was not considered severe. Since undersampling could lead to information loss and oversampling could increase the risk of overfitting in a relatively limited dataset [87], the original class distribution was preserved during the experiments. This full-sample class distribution was used as descriptive context, while the validation-specific majority-class baseline was used as the direct benchmark for interpreting model accuracy.

The forecasting results were presented and evaluated using three performance metrics: best accuracy, overall accuracy, and F1 scores. Given the observed full-sample class distribution of 78 positive and 50 negative purchase-decision observations, a majority-class classifier would achieve approximately 60.9% accuracy at the full-sample level. However, because the models were evaluated using fixed train–test partitions and repeated validation, the validation-specific majority-class baseline provides the appropriate benchmark for interpreting model accuracy. In this study, this validation-specific baseline was 53.85%. Therefore, the reported accuracy values are interpreted in relation to this benchmark rather than only in relation to the full-sample class distribution or a random-binary baseline. Accuracy is defined as the percentage of correct predictions, both true positives and true negatives, made by the model in relation to the total number of predictions made by the model [88]. Best accuracy refers to the highest accuracy value obtained from a model throughout the repeated validation process. Overall accuracy, on the other hand, refers to the mean accuracy value obtained across the forecasting procedure. It has been demonstrated that the best accuracy metric can be deceptive when employed in isolation, as it presents a single value and overlooks the broader performance of the model. In particular, Valverde-Albacete and Peláez-Moreno [89] demonstrate that classification accuracy can fail to capture crucial aspects of model performance, even when it is high, indicating that high accuracy does not necessarily imply better predictive capability. Therefore, it is employed as a form of supplementary information to facilitate the evaluation of overall accuracy, thereby addressing all the results obtained within the forecasting procedure. The F1 score is a metric used to measure the predictive power of a model, with higher scores indicating better predictive ability. F1 score is calculated as the harmonic mean of precision, defined as “the proportion of positive predictions that are correct”, and recall, which signifies the “percentage of actual positive cases that are correctly identified” by a particular model [88]. The details of the datasets and model results are explained below:

***Dataset 1.*** The initial forecasting results were obtained using 11 (fixation counts of 10 AOIs and scenario information) features and 1 target. The scenarios were incorporated into the models as categorical features. The output is the target, which represents the participant’s decision to purchase the product. After the models were trained, the participants’ decisions were forecasted using six algorithms. The accuracy values of the initial forecasts, representing the accurate forecasts out of 100%, are presented in Table 2. In forecasting with the fixation count metrics of all participants for 10 AOIs, and information on scenarios (1–4), SVM has shown the best overall accuracy of 60%, and the second-highest overall accuracy (58%) occurred with Logistic Regression. The best accuracy metrics reached up to 92% with this dataset regarding the ANN, SVM, Naive Bayes, and Logistic Regression methods. Random Forest was found to be the worst performer (51%) in both accuracy metrics. These results indicate that SVM and Logistic Regression achieved the strongest classification performance among the algorithms evaluated for fixation-count-based features.

***Dataset 2.*** Following the initial results, the fixation counts feature was replaced with another fixation metric, namely fixation durations. The data comprises 11 inputs (fixation durations of 10 AOIs and scenario information) and 1 target (purchase decision). The model training was repeated, and the accuracies given in Table 3 were attained. These findings suggest that fixation duration alone did not provide a clear improvement over fixation-count-based features in the present forecasting setting.

The substitution of the fixation count feature with fixation duration resulted in little to no enhancement of the overall accuracy of the Naïve Bayes, ANN, and Random Forest methods, while the remaining methods showed a slight decrease when compared with the findings of the preceding analysis with Dataset 1. On the other hand, the best accuracy of the ANN, SVM, Naïve Bayes, and Logistic Regression models declined compared with Dataset 1, while the Random Forest model remained unchanged. The highest best accuracy (92%) was observed in KNN. SVM retained the highest overall accuracy in Dataset 2, at 59%, which was slightly lower than in Dataset 1. Similarly, the Random Forest algorithm exhibited the lowest overall accuracy, as previously observed.

***Dataset 3.*** The final dataset comprises a full dataset of 21 inputs (fixation counts of 10 AOIs, fixation durations of 10 AOIs, and scenario information) and 1 output (purchase decision). As with the previous datasets, the categorical feature was taken as scenarios (1 to 4) assigned to participants. The training and testing procedures were repeated. The accuracy results are presented in Table 4. Dataset 3 provided comparable overall accuracy to Dataset 1, with slight improvements for ANN, Random Forest, KNN, and Logistic Regression. However, Random Forest still yielded the lowest overall accuracy among the evaluated methods (see Table 2, Table 3 and Table 4).

Table 5 summarizes the accuracy measures over three datasets, with the reported ranges showing the minimum and maximum values observed for each method. A consistent ranking pattern is observed in terms of overall accuracy, with Support Vector Machine (SVM) exhibiting the highest average accuracy across the datasets, achieving an overall accuracy of around 59–60%, while the Random Forest consistently yields the lowest results. Moreover, the differences in overall accuracy among the methods are relatively small; the best accuracy ranges largely overlap across methods, suggesting that this measure alone offers limited discriminatory value for forecasting performance.

To further address the interpretation of model accuracy, Support Vector Machines (SVM), which achieved the highest overall accuracy, and Logistic Regression, included as an interpretable benchmark model, were compared with the validation-specific majority-class baseline. The results are presented in Table 6. The complete model-level comparison for all algorithms is provided in Appendix A.3.

As shown in Table 6, SVM consistently achieved the highest overall accuracy and exceeded the validation-specific majority-class baseline by 5.15 to 6.15 percentage points across the three datasets. Logistic Regression also outperformed the baseline in all datasets, with improvements ranging from 2.15 to 5.15 percentage points. These results indicate that SVM and Logistic Regression, particularly SVM, provided a modest improvement over the naive majority-class benchmark.

The forecasting results show that the three datasets, which integrate different review-related AOI-based features with scenario information, yielded modest but informative classification outcomes. Fixation counts with scenarios (Dataset 1) achieved one of the highest overall accuracy levels observed in the study and yielded results comparable to those of the combined feature set, suggesting that fixation counts may contain useful behavioral information for purchase-decision classification. When fixation durations were considered separately (Dataset 2), the outcomes indicated limited additional forecasting value. Combining fixation counts and durations with scenarios (Dataset 3) yielded similar performance, with a slight increase for Logistic Regression, but did not substantially surpass the best levels observed with fixation counts alone. These results suggest that, within the scope of this research, fixation counts play a central role in forecasting accuracy, while fixation durations may provide complementary information when combined with fixation counts.

Regarding the methods, Artificial Neural Networks, Logistic Regression, and Naive Bayes attained the highest accuracy values in some validation runs, reaching up to 92%. However, these peak values should be interpreted cautiously, as the overall accuracy provides a more stable indicator of model performance. In terms of overall accuracy, Support Vector Machines achieved the highest average performance across all datasets. Logistic Regression also achieved competitive results, ranking second across all datasets. Random Forest, on the other hand, consistently yielded lower performance across datasets, making it the least favorable approach in this context.

As an additional diagnostic check, confusion matrices for selected best-performing model runs are provided in Appendix A.4. These matrices illustrate the models’ classification behavior for purchase and non-purchase decisions under selected validation runs.

## 5. Discussion

***Theoretical Implications:*** Our eye-tracking experiment provides insights into how individuals process product reviews in an online shopping environment, revealing that fixation patterns differ notably based on whether the motive was hedonic or utilitarian and on whether the product was intended for personal use or for others. This experimental observation is theoretically substantiated by the Review Helpfulness Theory, which asserts that the perceived helpfulness of reviews is not solely determined by objective review characteristics such as length or valence; rather, it is also influenced by the extent to which the content of the review corresponds with the informational requirements and motives of the reader [42]. This finding is consistent with Chen et al. [41] stating that “*consumers not only assess review helpfulness based on their consumption goals, but they also pay more attention (longer fixation duration and more fixation counts) to review the information that is consistent with their expectations*”. We observed that the participants tended to focus more on product reviews that matched their consumption motives, as reflected in longer fixation patterns. This observation matches the findings of Huang et al. [90] affirming that “*consumers were more likely to be involved in a deep comprehension of the reviews under match conditions*”. It is possible to associate the current research finding with that of Luan et al. [43], observing that the review type, namely attribute-based review (implying utilitarian motives) and experience-based review (implying hedonic motives), *“has a salient influence on consumers’ ocular behavior during their online shopping processes”.* Furthermore, we detected that when purchasing for others, they gave additional attention to hedonic keywords alongside nutritional values, regardless of motive type. Those in hedonic scenarios focused more on product appearance, and younger participants showed longer fixation durations overall.

These findings further extend Review Helpfulness Theory by adding an eye-tracking-based behavioral perspective to the processing of online reviews. While previous studies have mainly examined perceived helpfulness through review characteristics and consumer–review fit, the present study shows how consumers visually engage with review information during the decision-making process. In particular, gaze behavior toward review-related AOIs provides behavioral evidence of which review cues consumers attend to and revisit before making a purchase decision. Therefore, the contribution of this study lies in complementing Review Helpfulness Theory with visual-attention evidence, showing not only which reviews are considered helpful, but also how consumers process them visually.

***Managerial Implications:*** The experiment and its findings outlined in the current research have several noteworthy implications that contribute to our understanding of consumer purchasing decisions. Below, we list key implications:

- In promoting a product that targets utilitarian motives, it is crucial to clearly show the benefits it will provide to consumers. For instance, for food products, nutritional values can be highlighted, preferably with numerical details.
- If a product targeting utilitarian motives is being purchased for others, marketing should subtly include elements that evoke hedonic motives. Example: Feeling special, product appearance.
- If a product targeting hedonic motives is being purchased for others, the marketing should not only highlight the hedonic aspects but also mention who the product can be bought for and where it can be used (e.g., friends, in school, during sports).
- Regardless of the product’s motive, if it has a positive impact on consumers’ health, this should be clearly shown. For instance, for food products, instead of “rich in protein”, “8.1 g of protein” can be specified.
- If the target audience of the product consists of younger consumers (such as age groups of 18–22 and 23–27), the marketing can feature attention-demanding elements for a longer duration. Our findings reveal that total durations of fixations for ages 18–22 and 23–27 are longer than for ages 28–32, 33–37, and 38–42.
- The heat map observations suggest product visual design and packing may be important in hedonic shopping contexts. Especially, visual design cues become more salient when the product is purchased for someone else. This implication is based on heat map-based attention patterns rather than machine-learning forecasting results, which were limited to review-related AOIs.

***Methodological Implications:*** We also show that review-related gaze data can be utilized to obtain exploratory forecasts for purchasing decisions (purchase or not). We observed that using fixation counts or the combination of fixation counts and durations increased forecasting accuracy compared to relying solely on fixation durations. One potential explanation for the stronger performance of fixation counts relative to fixation durations is that fixation counts may more effectively capture repeated checking and comparison of review-related information. In the context of reading online reviews, consumers may revisit specific reviews or review cues multiple times as they form a purchase decision. By contrast, fixation duration indicates longer processing time, but it may also reflect hesitation, cognitive effort, or difficulty in interpreting information. Consequently, fixation counts may provide a more stable behavioral signal for purchase-decision classification than fixation duration alone.

Out of six machine-learning methods employed (*Artificial Neural Networks, Random Forest, Support Vector Machine, K-Nearest Neighbors, Naive Bayes, and Logistic Regression*), SVM achieved the highest overall accuracy (around 60%). Lowest accuracy metrics were observed for the Random Forest method. Overall, these findings indicate that review-related eye-tracking metrics can provide modest predictive value when evaluated against a validation-specific majority-class baseline. More importantly, the forecasting phase demonstrates the methodological feasibility of transforming objectively recorded gaze metrics into behavioral input features for machine-learning-based purchase-decision classification. From this perspective, the contribution of the forecasting component lies not only in the observed accuracy values, but also in showing how review-related visual attention indicators can be operationalized within a consumer behavior forecasting framework. Such data may also offer useful behavioral insight for marketers seeking to tailor strategies to consumer behavior more effectively. The results also point to several key points that should be considered when using such gaze data for forecasting consumer behavior:

- It is important to note that the results obtained from gaze-based metrics may vary depending on the methodology and experimental design employed. Different types of gaze data and eye-tracking metrics may contribute differently to forecasting outcomes. In addition, the modest accuracy levels observed in this study suggest that review-related gaze metrics should be regarded as one source of behavioral information, rather than as standalone predictors of purchase decisions.

Consumer behavior is a multidimensional phenomenon influenced by a wide range of factors. Within this context, the use of binary decision alternatives (purchase and do not purchase) to infer behavioral patterns reveals several methodological limitations. While this approach offers analytical simplicity, it limits the ability to capture states of uncertainty or hesitation that are common in consumer purchasing decisions. Expanding decision alternatives to reflect these latent decision states may lead to more robust behavioral representations, improve forecasting precision, and provide a promising avenue for future research.

## 6. Conclusions

The current research aims to enhance understanding of consumer purchasing decisions by examining eye-tracking data in relation to consumption motives (hedonic and utilitarian) and purchase purposes (purchasing for oneself and purchasing for others), while also providing insight into the forecasting of purchase decisions. The experimental design consists of several scenarios of purchasing that differentiate between motives and purchasing purposes. The purchase item was a chocolate bar from a fictitious brand with a specially designed package to avoid potential familiarity bias. After identifying several patterns regarding how eye-tracking data relate to varying consumption motives and varying purchase purposes, six machine-learning methods were used to analyze review-related gaze data for forecasting purchase decisions.

The eye-tracking results reveal that consumer attention is highly selective, with fixation patterns aligning closely with specific consumption motives and purchase purposes. Specifically, we observe that purchasing for others triggers increased attention toward hedonic attributes, even in utilitarian contexts, and that younger demographics exhibit significantly longer fixation durations. In the forecasting phase, SVM achieved the highest overall accuracy, approximately 59–60% across the evaluated datasets, and exceeded the validation-specific majority-class baseline by 5.15 to 6.15 percentage points. These findings suggest that review-related fixation metrics can provide modest predictive value for purchase-decision classification. More broadly, the forecasting results demonstrate how objectively recorded review-related gaze metrics can be operationalized as behavioral input features within a machine-learning-based consumer behavior forecasting framework. However, the forecasting model was intentionally limited to review-related AOIs and therefore does not capture the full range of visual stimuli, such as product image or packaging, that may also influence online purchase decisions. Collectively, these findings contribute to the literature by moving beyond descriptive analysis, and establishing a predictive framework that utilizes review-related gaze data to forecast purchase decisions.

The current research has several limitations that also suggest avenues for future research. First of all, in line with the objective of differentiating between motives and purchase purposes, we used a single fast-moving consumer product in our eye-tracking experiment. Future experiments may involve different product types, including high-involvement products such as luxury items, to examine whether product type influences visual attention and purchase decisions. Secondly, in our scenarios of “purchasing for someone else”, we prefer a general “another person”. The impact of the closeness of the relationship of the decision maker with the person the decision is made for can also be a factor to be analyzed. Finally, another factor to be analyzed in such a research design would be differences between demographically identical groups of participants. Demographic heterogeneity (such as age, gender, and occupation) may create variation in expectations and in the visual attention allocated to different parts of the provided information [91]. Although participants may ultimately arrive at similar decisions, this diversity may affect the generalizability of forecasting models [92].

A further limitation concerns the scope of the forecasting analysis. Although SVM and Logistic Regression outperformed the validation-specific majority-class baseline, the magnitude of improvement remained modest. This may be partly associated with the relatively homogeneous participant pool, the single-product context, and the binary purchase/no-purchase outcome, which may have limited the behavioral variability available for classification. Future research should test the proposed framework with larger and more heterogeneous samples, different product categories, alternative AOI definitions, and additional behavioral indicators to further examine the predictive contribution of eye-tracking metrics.

In addition to these limitations, the forecasting model’s scope should be considered. The machine-learning analysis was restricted to review-related AOIs as the study aimed to examine whether visual attention to user-generated reviews can provide predictive information about purchase decisions. Product image, packaging, price, brand, and other webpage elements were not included as predictive features. Therefore, the forecasting results should not be interpreted as representing the full visual decision-making process on an online shopping page. Future research may extend the framework by incorporating product image, packaging, and other visual AOIs to capture a more holistic representation of consumer visual attention.

Another limitation is the static online shopping interface and fixed 50 s exposure period. This design was selected to standardize visual exposure and isolate the effects of consumption motive and purchase purpose on attention to user-generated product reviews. However, this controlled setting differs from natural e-commerce browsing, where consumers may scroll through product information and reviews, selectively read or skip reviews, or leave the page quickly when their expectations are not met. In the present study, participants viewed a single product page with a fixed set of reviews. The design did not include product comparisons, navigation across multiple pages, or interactive browsing. Therefore, the findings should be interpreted within the boundaries of a controlled eye-tracking experiment focused on review reading. Future research may extend the present design by using interactive review interfaces, scrolling behavior, dynamic visual search tasks, multiple product alternatives, and clickstream data to better approximate real online shopping environments.

Overall, the current research offers a step forward in utilizing biometric data to understand consumer attention during review-based decision-making and provides a methodological basis for integrating eye-tracking indicators into consumer behavior forecasting, while opening avenues for further refinement in neuromarketing techniques.

## Figures and Tables

**Table 1:** Participant profile.

	Total Participants	Gender		Age Group				
		Female	Male	18–22	23–27	28–32	33–37	38–42
**Scenario 1**	32	20	12	19	9	1	2	1
**Scenario 2**	32	22	10	22	8	0	2	0
**Scenario 3**	32	23	9	21	8	3	0	0
**Scenario 4**	32	17	15	23	5	1	1	2

**Table 2:** Accuracy measures for the Dataset 1 forecasts with different methods.

Methods	ANN	Random Forest	SVM	KNN	Naive Bayes	Logistic Regression
**Overall Accuracy**	55%	51%	60%	56%	52%	58%
**Best Accuracy**	92%	85%	92%	85%	92%	92%
**F1 Score**	0.96	0.91	0.95	0.89	0.94	0.95

**Table 3:** Accuracy measures for the Dataset 2 forecasts with different methods.

Methods	ANN	Random Forest	SVM	KNN	Naive Bayes	Logistic Regression
**Overall Accuracy**	55%	51%	59%	54%	55%	56%
**Best Accuracy**	85%	85%	85%	92%	85%	85%
**F1 Score**	0.89	0.90	0.90	0.95	0.89	0.91

**Table 4:** Accuracy measures for the Dataset 3 forecasts with different methods.

Methods	ANN	Random Forest	SVM	KNN	Naive Bayes	Logistic Regression
**Overall Accuracy**	57%	52%	60%	55%	52%	59%
**Best Accuracy**	92%	77%	85%	85%	92%	92%
**F1 Score**	0.94	0.84	0.91	0.89	0.94	0.95

**Table 5:** Summary of accuracy measures over 3 datasets.

Methods	ANN	Random Forest	SVM	KNN	Naive Bayes	Logistic Regression
**Overall Accuracy**	55–57%	51–52%	59–60%	54–56%	52–55%	56–59%
**Best Accuracy**	85–92%	77–85%	85–92%	85–92%	85–92%	85–92%

**Table 6:** Baseline-adjusted comparison of SVM and Logistic Regression results.

Dataset	Validation-Specific Majority-Class Baseline	SVM Overall Accuracy	SVM Difference from Baseline (Percentage Points)	Logistic Regression Overall Accuracy	Logistic Regression Difference from Baseline (Percentage Points)
**Dataset 1**	53.85%	60%	+6.15	58%	+4.15
**Dataset 2**	53.85%	59%	+5.15	56%	+2.15
**Dataset 3**	53.85%	60%	+6.15	59%	+5.15

Note: Because the same train–test partitions were used across all machine-learning algorithms, the validation-specific majority-class baseline was identical across models within each dataset.

**Figure A1:**
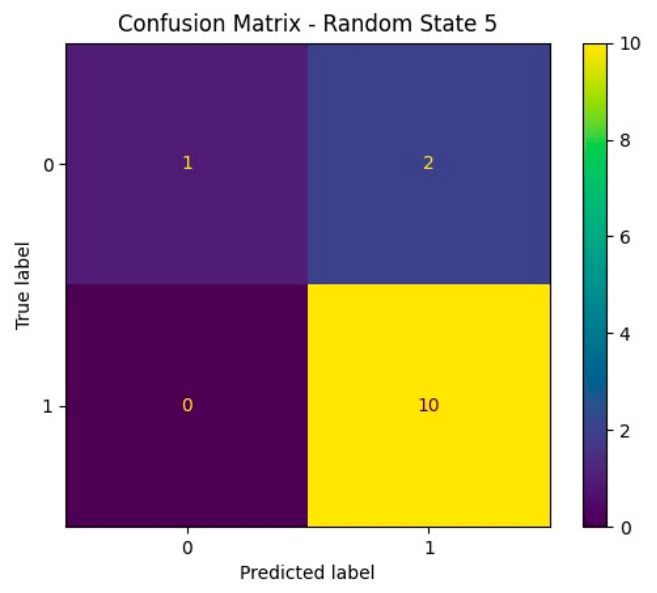
Confusion matrix for the Support Vector Machine model under Random State 5.

**Figure A2:**
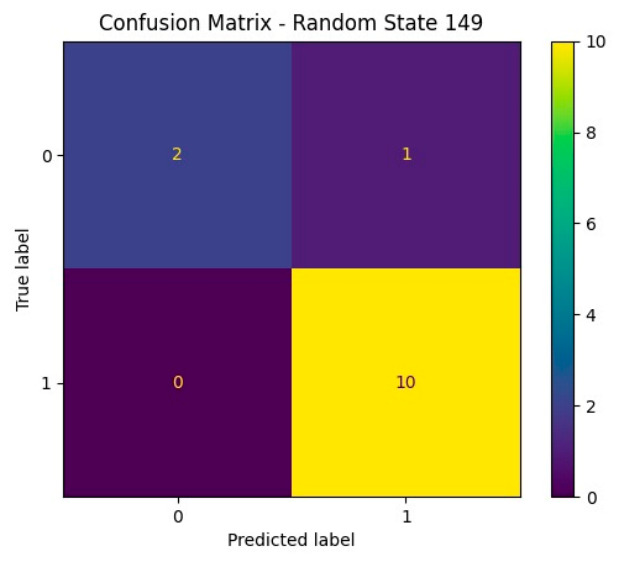
Confusion matrix for the Logistic Regression model under Random State 149.

## Data Availability

The data and codes used in this study are publicly available at: https://github.com/badslab-collab/consumption-motives (accessed on 30 May 2026).

## Abbreviations

The following abbreviations are used in this manuscript:

AOI	Areas of Interest
ANN	Artificial Neural Network
SVM	Support Vector Machine
KNN	K-Nearest Neighbor
F1 Score	F1 Score of Best Accuracy

## Appendix A

### Appendix A.1

**Table A1:** *t*-test results for scenarios.

Utilitarian items	Scenario 1	Scenario 2	Scenario 3	Scenario 4
Scenario 1	-	0.874	0.000 *	0.000 *
Scenario 2	0.874	-	0.000 *	0.000 *
Scenario 3	0.000 *	0.000 *	-	
Scenario 4	0.000 *	0.000 *	0.978	-
Hedonic items				
Scenario 1	-	0.855	0.039 *	0.018 *
Scenario 2	0.855	-	0.024 *	0.011 *
Scenario 3	0.039 *	0.024 *	-	0.585
Scenario 4	0.018 *	0.011 *	0.585	-

* Significant at 95%.

### Appendix A.2

**Table A2:** Translation of reviews.

***Utilitarian reviews***
Compared to other products *ChocoPist* provides more energy. When you look at the 571 kcal amount you can see that clearly. Thanks to the 8.1 g of protein it contains, one pack assuages your hunger. It’s a bit high in fats but it contains no trans-fat. Such a price-performance product. It is very good for balancing blood sugar and raising your energy. Even though the sugar amount is high, the real pistachio content makes it rich for vitamins and minerals. I found that the amount of cream inside is low. It does not provide enough nutritional value but in emergencies it is lifesaving!
***Hedonic reviews***
With its unique style which makes one think of handmade chocolates, *ChocoPist* is indispensable to those who want to make themselves and (lucky) others special! It is very obvious that packaging was made sloppy! All the product came out to be broken. Even though it was all broken the taste was legendary. The visual and the packaging is beautiful but there is an artificial pistachio taste. Even it didn’t meet my expectation my children love it! *ChocoPist* is a wonderful snack that can accompany you; at your home, on the way, at the school or office, basically everywhere. Generally, I do not enjoy a chocolate waffle but after very strong recommendations from my friends, I bought one. Unfortunately, it did not give me the same pleasure.

### Appendix A.3

**Table A3:** Model-level comparison with the validation-specific majority-class baseline. The validation-specific majority-class baseline was 53.85% for all models because the same train-test partitions were used across all machine-learning algorithms to ensure fair comparison and reproducibility. Therefore, the baseline is reported once, and the table presents each model’s overall accuracy together with its difference from this baseline. Difference values are expressed in percentage points.

Dataset	Model	Overall Accuracy	Difference from Baseline
**Dataset 1**	ANN	55%	+1.15
**Dataset 1**	Random Forest	51%	−2.85
**Dataset 1**	SVM	60%	+6.15
**Dataset 1**	KNN	56%	+2.15
**Dataset 1**	Naive Bayes	52%	−1.85
**Dataset 1**	Logistic Regression	58%	+4.15
**Dataset 2**	ANN	55%	+1.15
**Dataset 2**	Random Forest	51%	−2.85
**Dataset 2**	SVM	59%	+5.15
**Dataset 2**	KNN	54%	+0.15
**Dataset 2**	Naive Bayes	55%	+1.15
**Dataset 2**	Logistic Regression	56%	+2.15
**Dataset 3**	ANN	57%	+3.15
**Dataset 3**	Random Forest	52%	−1.85
**Dataset 3**	SVM	60%	+6.15
**Dataset 3**	KNN	55%	+1.15
**Dataset 3**	Naive Bayes	52%	−1.85
**Dataset 3**	Logistic Regression	59%	+5.15
